# Hyperplastic Human Macromass Cartilage for Joint Regeneration

**DOI:** 10.1002/advs.202301833

**Published:** 2023-07-03

**Authors:** Ya Wen, Yishan Chen, Weiliang Wu, Hong Zhang, Zhi Peng, Xudong Yao, Xianzhu Zhang, Wei Jiang, Youguo Liao, Yuan Xie, Xilin Shen, Heng Sun, Jiajie Hu, Hua Liu, Xiao Chen, Jiansong Chen, Hongwei Ouyang

**Affiliations:** ^1^ Dr. Li Dak Sum & Yip Yio Chin Center for Stem Cells and Regenerative Medicine and Department of Orthopedic Surgery of the Second Affiliated Hospital Zhejiang University School of Medicine Hangzhou 310058 China; ^2^ Department of Sports Medicine Zhejiang University School of Medicine Hangzhou 310058 China; ^3^ Zhejiang University‐University of Edinburgh Institute Zhejiang University School of Medicine and Key Laboratory of Tissue Engineering and Regenerative Medicine of Zhejiang Province Zhejiang University School of Medicine Hangzhou 310058 China; ^4^ Department of Orthopedic Surgery The Children's Hospital Zhejiang University School of Medicine National Clinical Research Center for Child Health Hangzhou 310052 China; ^5^ The Fourth Affiliated Hospital International Institutes of Medicine Zhejiang University School of Medicine Yiwu 322000 China; ^6^ Department of Orthopedics The First Affiliated Hospital, Zhejiang University School of Medicine Hangzhou 310003 China; ^7^ Department of Orthopaedic Surgery Orthopaedic Institute The First Affiliated Hospital Medical College Soochow University Suzhou 215006 China; ^8^ China Orthopedic Regenerative Medicine Group (CORMed) Hangzhou 310058 China

**Keywords:** cell plasticity, customized expansion, joint regeneration, macromass cartilage

## Abstract

Cartilage damage affects millions of people worldwide. Tissue engineering strategies hold the promise to provide off‐the‐shelf cartilage analogs for tissue transplantation in cartilage repair. However, current strategies hardly generate sufficient grafts, as tissues cannot maintain size growth and cartilaginous phenotypes simultaneously. Herein, a step‐wise strategy is developed for fabricating expandable human macromass cartilage (macro‐cartilage) in a 3D condition by employing human polydactyly chondrocytes and a screen‐defined serum‐free customized culture (CC). CC‐induced chondrocytes demonstrate improved cell plasticity, expressing chondrogenic biomarkers after a 14.59‐times expansion. Crucially, CC‐chondrocytes form large‐size cartilage tissues with average diameters of 3.25 ± 0.05 mm, exhibiting abundant homogenous matrix and intact structure without a necrotic core. Compared with typical culture, the cell yield in CC increases 2.57 times, and the expression of cartilage marker collagen type II increases 4.70 times. Transcriptomics reveal that this step‐wise culture drives a proliferation‐to‐differentiation process through an intermediate plastic stage, and CC‐chondrocytes undergo a chondral lineage‐specific differentiation with an activated metabolism. Animal studies show that CC macro‐cartilage maintains a hyaline‐like cartilage phenotype in vivo and significantly promotes the healing of large cartilage defects. Overall, an efficient expansion of human macro‐cartilage with superior regenerative plasticity is achieved, providing a promising strategy for joint regeneration.

## Graphical abstract

By using a customized culture for human polydactyly chondrocytes, an efficient expansion of large‐size human cartilage analogs (macro‐cartilage) exhibited with abundant cartilage extracellular matrix is achieved. This study proposes an encouraging avenue for the fabrication of off‐the‐shelf cartilage grafts addressing both quantity and quality demands, and also provides new insights into the understanding of the cell‐intrinsic regenerative potential.

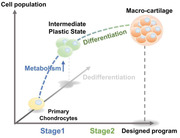

## 1 Introduction

Articular cartilage is vital for the lubrication and cushioning of joint movements. However, the human adult articular cartilage possesses poor self‐healing ability and only contains a limited number of chondrocytes, which occupy less than 5% of the whole tissue volume.^[^ ^1^ ^]^ Without proper treatment, an accidental cartilage injury may lead to the onset of osteoarthritis (OA), a disease that afflicts more than 16% of adults and costs nearly US$7000 per patient‐year.^[^ ^2^ ^]^

Cartilage is an immune‐privileged tissue, so engineered cartilage analogs can serve as alternative off‐the‐shelf grafts in allogeneic transplantation.^[^ ^3^ ^]^ These small, 3D cultures are designed to recapitulate the in vivo physiology and resemble natural tissues.^[^ ^4^ ^]^ Compared to autologous chondrocyte implantation (ACI), cartilage analog transplantation adopts well‐standard grafts for patients who lack healthy chondrocytes.^[^ ^5^ ^]^ Without an additional invasive operation to obtain cells, it makes the surgical procedure faster and postoperative management simpler.^[^ ^6^ ^]^ However, current techniques still show limitations in the generation of ideal tissues with both robust proliferation and stable lineage function. For example, the chondrocytes phenotype can be well maintained in a 3D microenvironment, but the proliferation is inhibited to some extent and results in an arrest in tissue growth.^[^ ^7^ ^]^ Thus, the improved volume of cartilaginous tissues relies on an increased input of healthy chondrocytes. When the tissue diameter exceeds 1.5 mm using the current methodology, it is difficult to obtain high‐quality homogeneous cartilage without necrotic cores.^[^ ^4^, ^8^ ^]^ Consequently, the expansion is compromised, only yielding small aggregations.^[^ ^7^, ^9^ ^]^ Therefore, it requires the development of new strategies to obtain expandable and functional cartilage analogs by using a highly functional cell resource and an optimal culture system.

Human polydactyly‐derived chondrocytes are an easily accessible resource because the prevalence of polydactyly is ≈0.3–3.6/1000 live births.^[^ ^10^ ^]^ Evidences have shown that polydactyly cells possess a high phenotype plasticity as they showed a superior proliferation compared to adult chondrocytes, and were capable of forming hyaline‐like cartilage.^[^ ^9^, ^11^ ^]^ However, the existing culture conditions, which adopt a serum‐containing medium, are not suitable for these applicable cells. It was documented that the serum‐containing medium might induce a loss of cellular function.^[^ ^12^ ^]^ The components in serum vary in batches and cannot be completely identified and controlled.^[^ ^13^ ^]^ Thus, a fully‐defined custom‐designed medium needs to be developed, which has been shown to improve the production of desirable tissues by regulating specific programs and is extensively applied in 3D cultures of many other cell types.^[^ ^13^, ^14^ ^]^ In particular, it is urgent to develop a tailor‐made culture strategy to maximize the potential of polydactyly chondrocytes for the fabrication of expandable functional human cartilage.

Here, we aimed to develop a cartilage analog to provide an off‐the‐shelf, high‐performance graft for cartilage injury. By factors screening, we obtained a customized culture (CC) condition that allows polydactyly chondrocytes to proliferate and retain lineage function. CC‐chondrocytes can also proliferate in a 3D environment, and form large‐size tissues with abundant cartilage extracellular matrix (ECM). RNA sequencing uncovered that CC drove a proliferation‐to‐differentiation with a metabolically active intermediate stage. Implantation of the macromass cartilage (macro‐cartilage) functionally facilitated the repair of large cartilage defects in a rat model. As a result, our findings provided a novel strategy for producing human macro‐cartilage, holding great potential as an alternative tissue source for large‐size cartilage defect repair.

## 2 Results

### 2.1 A Screen‐Defined Serum‐Free Medium Efficiently Expands Human Chondrocytes

To obtain chondrocyte resources, polydactyly cartilage was isolated from juvenile patients around 1 year old (Table S1, Supporting Information). The polydactyly cartilage exhibited a hyaline cartilage‐like phenotype with intense Safranin‐O (SO) staining (Figure S1A, Supporting Information). To establish a serum‐free condition optimal for human chondrocytes, we conducted a primary screen for essential supplements using DMEM/F12 as the basic medium.^[^ ^11^, ^15^ ^]^ Assuming that the optimal condition should induce the proliferation of juvenile chondrocytes while maintaining their lineage phenotype, we selected transforming growth factor beta‐3 (TGF*β*3) as a candidate supplement, because it played an important role in cartilage development and homeostasis.^[^ ^16^ ^]^ Basic fibroblast growth factor (bFGF) and B27 supplement were also adopted since they were shown to support the growth of stem cells,^[^ ^17^ ^]^ which might be suitable for juvenile cells.

**Figure 1:**
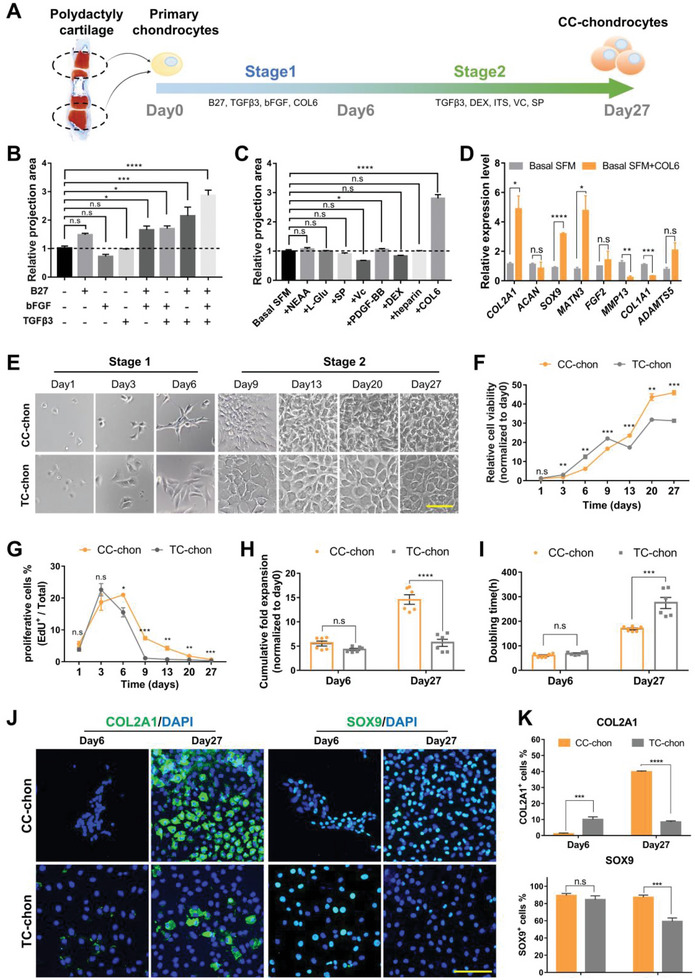
The customized culture enables chondrocytes to proliferate and also express lineage markers. A) Schematic overview of the designed program for step‐wise culture. B) The contribution of different combinations of elements to chondrocytes expansion (*n* = 3, one‐way ANOVA followed by Dunnett's multiple comparison test). C) The contribution of different additives to the optimization of expansion (*n* = 3, one‐way ANOVA followed by Dunnett's multiple comparison test). D) Gene expression analysis of chondrocytes markers (COL2A1, ACAN, SOX9, MATN3, FGF2, MMP13, COL1A1 and ADAMTS5) in chondrocytes cultured with/without COL6 (*n* = 3, unpaired two‐tailed Student's *t*‐tests). E) Representative images of chondrocytes cultured in CC and TC over time. Scale bars: 100 µm. F) Cell viability of CC‐chons and TC‐chons measured by CCK‐8 kit over time (*n* = 3, unpaired two‐tailed Student's *t*‐tests). G) Percentage of proliferating cells (EdU^+^) in CC and TC over time (*n* = 3, unpaired two‐tailed Student's *t*‐tests. H) Cumulative fold expansion at day 6 and day 27, normalized to day 0 (*n* = 6, unpaired two‐tailed Student's *t*‐tests). I) Doubling time of chondrocytes cultured in CC and TC (*n* = 6, unpaired two‐tailed Student's *t*‐tests). J) Representative images of immunofluorescent staining of chondrocyte markers (COL2A1 and SOX9) in CC‐chons and TC‐chons. Scale bars: 100 µm. K) Efficiency quantification of COL2A1 and SOX9 staining in CC‐chons and TC‐chons at day 6 and day 27, respectively (*n* = 6, unpaired two‐tailed Student's *t*‐tests). All data were mean ± SEM. n.s *p* ≥ 0.05, *p < 0.05, **p < 0.01, ***p < 0.001, ****p < 0.0001.

Although the petri‐dish surface benefits cell adhesion and spreading, we still found that the polydactyly chondrocytes spontaneously formed small cell clusters when they were cultured in an appropriate medium (Figure S2A,B, Supporting Information). The area of cell clusters was used to quantify cell growth. Our data showed that adding B27, bFGF, and TGF*β*3 (Table S2, Supporting Information) alone did not affect cell proliferation. The combination of those two or three promoted the formation and expansion of cell clusters. This effect was further maximized in the combination of the B27, bFGF, and TGF*β*3 (**Figure** **1**B and Figure S2A, Supporting Information). Following by using DMEM/F12 plus B27, bFGF, and TGF*β*3 as the basal serum‐free medium (basal SFM), we screened another eight additives that were highly relevant to chondrogenic induction for further optimization. These additives were MEM non‐essential amino acids (NEAA), l‐glutamine (l‐Glu), sodium pyruvate (SP), ascorbic acid (Vc), platelet‐derived growth factor‐BB (PDGF‐BB), dexamethasone (DEX), heparin sodium (Heparin), and collagen type VI (COL6) (Table S3, Supporting Information).^[^ ^12^, ^18^ ^]^ COL6, a major component of the chondrocyte pericellular matrix, stimulated the proliferation of chondrocytes in our results (Figure 1C and Figure S2B, Supporting Information), which was consistent with a previous study.^[^ ^18b^ ^]^ Moreover, the addition of COL6 upregulated some cartilage markers (e.g., collagen type II alpha 1 chain (*COL2A1*), SRY‐box 9 (*SOX9*), matrilin 3 (*MATN3*)) (Figure 1D).^[^ ^17^, ^19^ ^]^ The effect of B27, bFGF, TGF*β*3, and COL6 on cell proliferation was verified by removing them separately from the mixture of all candidates (Figure S2C,D, Supporting Information).

In conclusion, we primarily established a customized serum‐free medium (CM, consisting of DMEM/F12, B27, bFGF, TGF*β*3, and COL6), which significantly accelerated the proliferation of polydactyly‐derived chondrocytes.

### 2.2 The Customized Culture Enables Chondrocytes to Proliferate and Also Express Lineage Markers

To obtain chondrocytes with sufficient chondrogenic features, we designed a step‐wise culture protocol consisting of a 6‐day proliferation and a 21‐day differentiation procedure (Figure 1A). Chondrocytes expanded in customized culture (CC‐chons) were characterized during or at the end of stages 1 (day 6) and 2 (day 27). Chondrocytes expanded in typical serum‐containing DMEM/F12 medium (TC‐chons) and served as controls.

Compared with the TC‐chon group, the CC‐chon group exhibited higher cell density, particularly in stage 2 (Figure 1E and Figure S3A,B, Supporting Information). The cell viability assay also demonstrated the sustained increase of total live cells in the CC‐chon group (Figure 1F). Using 5‐ethynyl‐2′‐deoxyuridine (EdU) to label proliferating cells, we discovered that the CC group had better‐proliferating chondrocytes than the TC group since day 3 (Figure 1G and Figure S3A, Supporting Information). At the end of stage 1 (day 6), comparable expansion was detected in both groups (5.53 ± 0.51‐fold in CC vs 4.31± 0.22‐fold in TC) (Figure 1H). However, after 21 days of chondrogenesis (day 27), the yield of CC‐chons reached 14.59 ± 0.99‐fold cumulatively, while TC‐chons were only expanded 5.68 ± 0.74‐fold (Figure 1H). In the 27‐days culture procedure, CC‐chons had a higher proliferation efficiency, and the average doubling time was only 61.53% of that of TC‐chons (168.83 ± 4.32‐h in CC vs 274.40 ± 22.44‐h in TC) (Figure 1I). These results indicated that CC induced a more robust proliferation.

To further characterize CC‐chons, we examined the expression of chondrocyte functional markers. Immunofluorescence revealed that both CC‐chons and TC‐chons exhibited low expression levels of COL2A1 at the end of stage 1 (day 6) (Figure 1J,K). However, after stage 2, the expression of COL2A1 increased significantly in CC‐chons (39.82 ± 0.53% in CC vs 8.48 ± 0.67% in TC), even though the condition (monolayer culture) was not fully appropriate for chondrogenesis (Figure 1J,K). Moreover, CC‐chons at both stages showed a high expression level of SOX9 (Figure 1J,K). Together, these results demonstrated that CC‐chons had a superior functional phenotype of cartilage lineage relative to TC‐chons. To check whether cells acquired a fibrotic phenotype, we also examined the expression of collagen type I (COL1). Cells in both groups expressed high levels of COL1 on day 6, and there was no significant difference between the two groups (Figure S3C,D, Supporting Information). Nevertheless, a notable decrease in COL1 expression was observed after the culture of stage 2 (day 27), indicating a recovery of the chondrocyte phenotype.

In brief, this section revealed that the customized step‐wise culture induced a robust proliferation at an early stage, and eventually gave rise to a typical chondrogenic phenotype in human chondrocytes.

### 2.3 CC‐Chons can Spontaneously Aggregate and Efficiently Proliferate in 3D Culture

**Figure 2:**
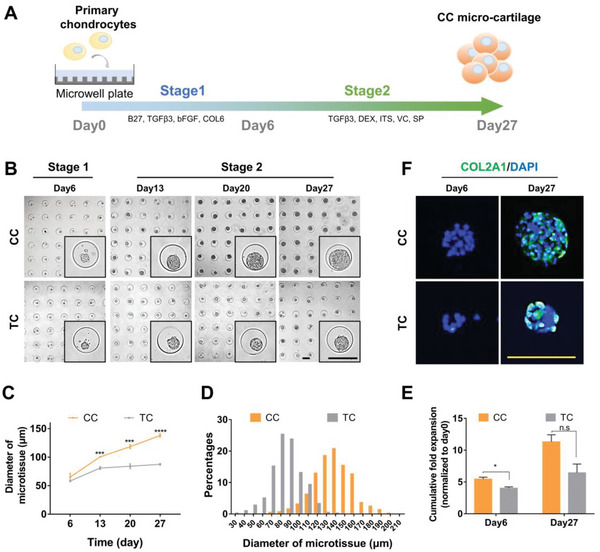
CC‐chons can spontaneously aggregate and efficiently proliferate in 3D culture. A) Schematic diagram illustrating the designed program for step‐wise scaling up of micro‐cartilage. B) Representative bright‐field images of CC and TC microtissues over time. Scale bars: 200 µm. C) Diameter of CC and TC microtissues over time (*n* = 3, unpaired two‐tailed Student's *t*‐tests). D) Frequency distribution of diameter of CC and TC microtissues on day 27 (*n*_CC_ = 448, *n*_TC_ = 395). E) Cumulative fold expansion of CC and TC treatments at day 6 and day 27 (*n* = 3, unpaired two‐tailed Student's *t*‐tests). F) Representative immunostaining images of COL2A1 in CC and TC microtissues. Scale bars: 200 µm. All data were mean ± SEM. n.s *p* ≥ 0.05, *p < 0.05, ***p < 0.001.

To test the effect in 3D expansion, we applied our customized culture in microtissue fabrication. We employed a high‐throughput microwell platform made of agarose because its non‐adhesive property promotes spontaneous cell aggregation on the surface (**Figure** **2**A).^[^ ^20^ ^]^ The agarose with microwells was punched into a 1.8 cm^2^ insert to fit in a well of a 24‐well plate. Each insert contained about 1000 individual microwells with a diameter of 200 µm, as previously described.^[^ ^7^, ^20^ ^]^

Freshly isolated primary chondrocytes were seeded directly into microwells at a density of 20 cells per microwell. They were cultured in the step‐wise formula given in Figure 1A (a 6‐day proliferation plus 21‐day chondrogenesis). Self‐aggregation was observed on day 6 (Figure 2B). Spherical microtissues were formed and increased in size over time (Figure 2B). On day 27, the average diameter of the CC microtissues reached 137.90 ± 1.59 µm, while that of TC microtissues was only 87.43 ± 0.87 µm (Figure 2C,D). Furthermore, cells in CC microtissues were expanded 11.31 ± 1.11‐fold relative to the initial number, which was 1.75 times that of TC microtissues (6.45 ± 1.37‐fold) (Figure 2E). These findings showed that CC initiated an enhanced proliferation in 3D expansion. Additionally, immunofluorescence staining revealed the expression of COL2A1 in day 27 microtissues, indicating the formation of cartilage ECM (Figure 2F).

In summary, although previous studies have shown that chondrocyte proliferation is restricted in a 3D environment,^[^ ^7^ ^]^ here we achieved an expansion of self‐organized cartilaginous microtissues (micro‐cartilage) with the desired size.

### 2.4 CC‐Chons Can Form Large‐Size and ECM‐Rich Macro‐Cartilage in 3D Culture

**Figure 3:**
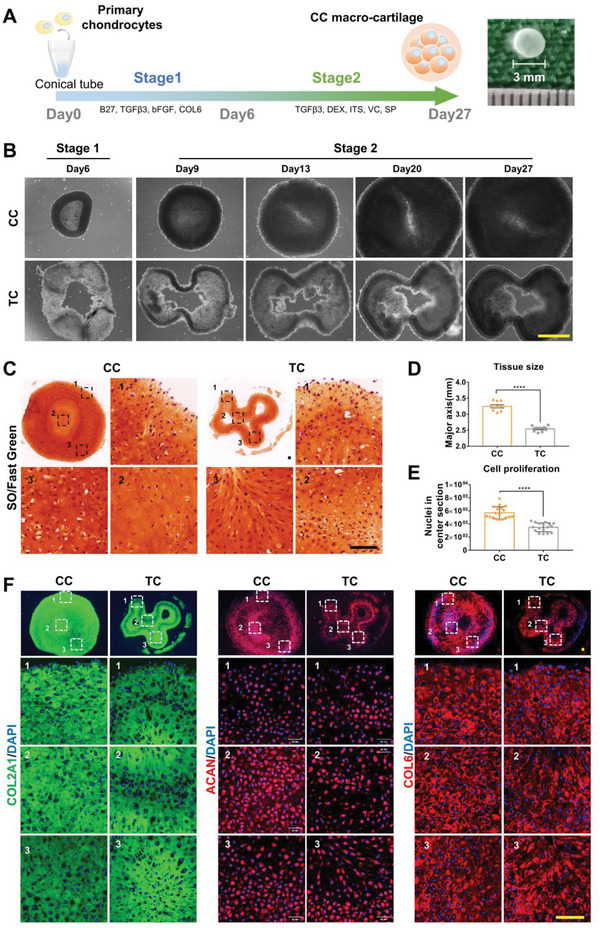
CC‐chons can form large‐size and ECM‐rich macro‐cartilage in 3D culture. A) Schematic diagram illustrating the designed program for step‐wise pellet culture. B) Representative bright‐field images of tissues by CC and TC incubation over time. Scale bar: 1 mm. C) SO/fast green staining of CC and TC tissues. Scale bar: 100 µm. D) Major axis quantification of center section in CC and TC tissues (*n* = 3, unpaired two‐tailed Student's *t*‐tests). E) Quantification of cell number in the central section (*n* = 3, unpaired two‐tailed Student's *t*‐tests). F) Representative images of immunofluorescent staining of chondrocyte markers (COL2A1, ACAN, and COL6) in CC and TC tissues. Scale bar: 100 µm. All data were mean ± SEM. ****p < 0.0001.

Due to methodological limitations, previous studies produced only small cartilage tissues, usually less than 1.5 mm in diameter.^[^ ^7^, ^9^ ^]^ To determine the capability of CC‐chons to form large‐size tissues, we also conducted typical pellet culture. 2 × 10^5^ freshly isolated chondrocytes were centrifuged in conical tubes with an inner diameter of 3 mm (**Figure** **3**A). The step‐wise change of medium was performed as illustrated in Figure 1A. Chondrocytes cultured in CC condensed into an intact disc‐like structure on day 6 (Figure 3B). These CC constructs enlarged significantly over time and their main axis reached 3.25 ± 0.05 mm on day 27 (Figure 3B–D). While chondrocytes cultured in TC showed poorer aggregation and formed an irregular ring‐like construct on day 6 (Figure 3B). Although the tissue volume increased, the hollow structures in TC showed no further aggregation, with a major axis of 2.54 ± 0.03 mm on day 27 (Figure 3B–D). The CC constructs outperformed the TC constructs in terms of proliferative capacity (Figure S4A, Supporting Information) and cellular content in the central ((5.70 ± 0.22) × 10^3^ in CC vs (3.49 ± 0.16) × 10^3^ in TC) (Figure 3E). Live/Dead staining and trypan blue staining results demonstrated that the majority of cells in the macromass tissues remained viable during the culture procedure (Figure S4B,C, Supporting Information). These data demonstrated that CC supported the formation and growth of macro‐tissue.

To assess the quality of macro‐tissues, we analyzed cartilage ECM biomarkers. Rich glycosaminoglycan (GAG) was presented in both groups by the intense SO staining (Figure 3C). CC and TC tissues both positively expressed articular cartilage biomarkers such as COL2A1, aggrecan (ACAN), COL6, and proteoglyacan 4 (PRG4) (Figure 3F and Figure S4D,E, Supporting Information). Notably, GAG and collagen proteins were distributed evenly in CC macro‐tissues, whereas there was an obvious regional heterogeneity in TC tissues (Figure 3F). In addition, high magnification images of standard error of the mean (SEM) demonstrated that tissues of both groups had an ECM structure with a smooth GAG‐rich surface and a few cable‐like collagen fibers (Figure S4F, Supporting Information).

These results illustrated that CC‐chons were capable of forming intact macro‐cartilage with homogeneous typical cartilage ECM.

### 2.5 Transcriptomics Reveals that the Customized Culture Induces a Lineage‐Specific Differentiation with Activated Metabolism

**Figure 4:**
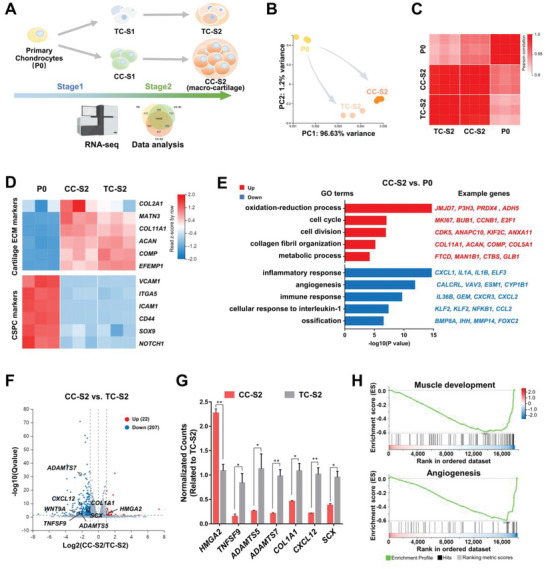
Transcriptomics reveals that the customized culture induces lineage‐specific differentiation with activated metabolism. A) Schematic diagram of transcriptomics workflow. B) Principal‐component analysis (PCA) plot of primary chondrocytes (P0) and end‐stage tissues (CC‐S2, TC‐S2). C) Correlation coefficient matrix of P0, CC‐S2, and TC‐S2. D) Heatmaps of CSPC markers and cartilage markers in P0, CC‐S2, and TC‐S2. E) Gene ontology (GO) enrichment analysis comparison between P0 and CC‐S2. F) Volcano plot identifying genes that are differentially expressed between CC‐S2 and TC‐S2. G) Relative expression of representative differentially expressed genes in CC‐S2 and TC‐S2 (*n* = 3, unpaired two‐tailed Student's *t*‐tests). H) Gene set enrichment analysis (GSEA) enrichment analysis comparison between CC‐S2 and TC‐S2. All data were mean ± SEM. *p < 0.05, **p < 0.01.

To analyze the overall transcriptome features of cultured cells and dissect the induction programs, we conducted RNA sequencing (RNA‐seq) on primary polydactyly chondrocytes (P0), TC and CC tissues at stages 1 and 2, respectively (CC‐S1, TC‐S1, CC‐S2 and TC‐S2, *n* = 3) (**Figure** **4**A).

The sample‐to‐sample comparison revealed that among the cells of the initial and end‐stage time points, the end‐stage tissues (CC‐S2, TC‐S2) were relatively similar to each other, but more distinct from the original primary chondrocytes (P0) (Figure 4B,C and Figure S5A,B, Supporting Information). CC‐S2 and TC‐S2 showed an enhanced expression of cartilage ECM genes (e.g., *COL2A1*, *MATN3*, *ACAN*, and cartilage oligomeric matrix protein (*COMP*)) compared with P0 (Figure 4D).^[^ ^19^, ^21^ ^]^ Considering that P0 was derived from infants, we also investigated the features of chondrogenic stem/progenitor cells (CSPCs). A higher level of CSPCs markers (e.g., vascular cell adhesion molecule 1 (*VCAM1*), intercellular adhesion molecule 1 (*ICMA1*), and notch receptor 1 (*NOTCH1*)) were highly expressed in P0, relative to both end‐stage tissues (Figure 4D).^[^ ^22^ ^]^ These differences indicated that both CC‐chons and TC‐chons were stimulated into a more differentiated state after step‐wise induction.

To further identify the state of macro‐cartilage, we projected our RNA‐seq data and published datasets of human articular cartilage at different developmental stages (GSE106292). In the princial‐component analysis (PCA) plot, we found that polydactyly‐derived cartilage tissues (P0, CC‐S2, TC‐S2) were clustered together, and exhibited a similar phenotype with fetal cartilage (17 weeks old) compared with adult cartilage (Figure S5C, Supporting Information). It indicated that the constructed macro‐cartilage resembled human naïve cartilage, and might explain their superior ability of proliferation and the potential of chondrogenic differentiation.

To compare the samples in detail, we analyzed the differentially expressed genes (DEGs) of CC‐S2 and P0. 3057 genes were up‐regulated in CC‐S2, while 2952 genes showed a higher expression in P0 (Figure S5D, Supporting Information). Gene ontology (GO) terms of both proliferation and cartilage ECM (cell cycle, cell division, and collagen fibril organization) were enriched in CC‐S2, supporting the heightened proliferative and chondrogenic features (Figure 4E). The relevant genes were *MKI67*, *BUB1*, *CCNB1*, *COL11A1*, *ACAN*, *COMP*, etc. It was also worth noting that genes related to the oxidation‐reduction process, and metabolic process were evidently increased in CC‐S2 (Figure 4E). On the contrary, genes associated with inflammatory response, angiogenesis, immune response, cellular response to interleukin‐1, and ossification were significantly repressed in CC‐S2 (Figure 4E). These results highlighted that CC‐S2 obtained a unique transcriptional feature when induced from primary polydactyly chondrocytes including a higher expression of proliferative, differentiated and metabolic genes, and a lower level of inflammatory markers.

We next compared the end‐stage tissues formed in two different conditions (CC‐S2 vs TC‐S2). In CC‐S2, 22 genes were shown with an enhanced expression, while 207 genes were down‐regulated (Figure 4F). In comparison to CC‐S2, the features in TC‐S2 were associated with inflammation (e.g., *TNFSF9*, *CXCL12*), ECM degradation (e.g., *ADAMTS5*, *ADAMTS7*), and cartilage fibrosis (e.g., *COL1A1*, *SCX*) (Figure 4F,G).^[^ ^23^ ^]^ *WNT9A*, an activator of the canonical WNT signaling that could inhibit chondrogenesis, was also relatively highly expressed in TC‐S2.^[^ ^24^ ^]^ Gene set enrichment analysis (GSEA) demonstrated that genes related to muscle development (e.g., myocyte enhancer factor 2B (*MEF2B*), striated muscle enriched protein kinase (*SPEG*), and interferon related developmental regulator 1 (*IFRD1*)) and angiogenesis (e.g., cadherin 13 (*CDH13*), epithelial mitogen (*EPGN*), and angiopoietin‐like 4 (*ANGPTL4*)) were significantly higher in TC‐S2 (Figure 4H). These results indicated that CC induced a more cartilage lineage‐specific phenotype when compared to a typical serum‐containing medium.

In a word, the RNA‐seq data revealed that CC culture drove a differentiation of primary juvenile chondrocytes with active metabolism and suppressed inflammation. By comparing CC‐S2 and TC‐S2 samples, we demonstrated that CC‐chons obtained a proliferative characteristic and less markers of other lineages.

### 2.6 The Step‐Wise Culture Drives a Proliferation‐to‐Differentiation Progress through an Intermediate Plastic Stage

**Figure 5:**
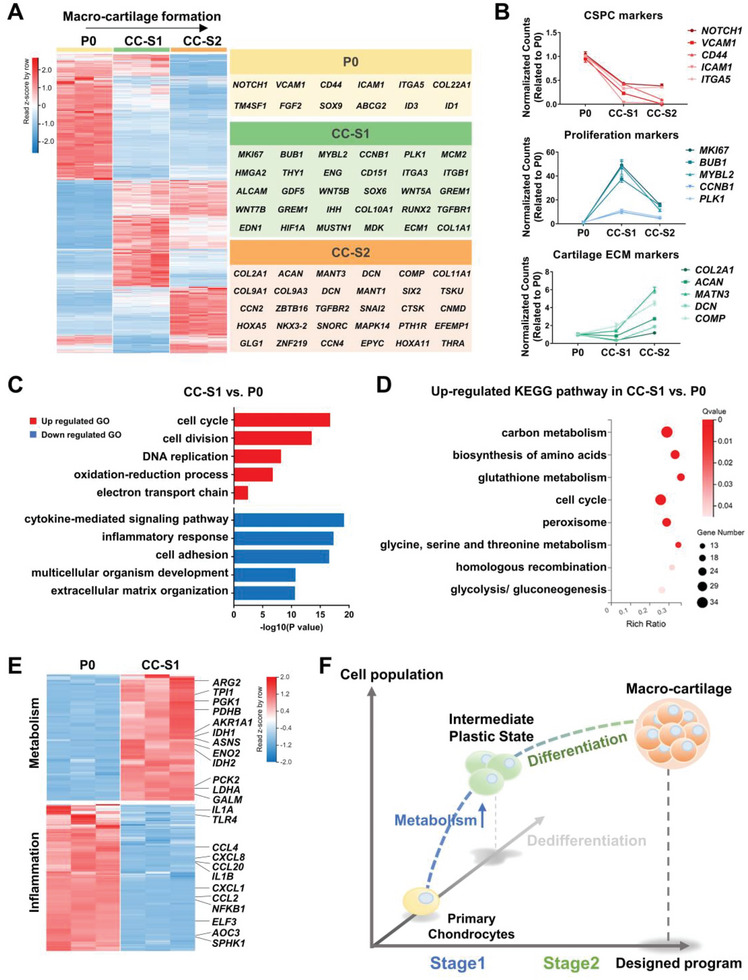
The step‐wise culture drives proliferation‐to‐differentiation progress through an intermediate stage. A) Heatmap of differentially expressed genes in P0, CC‐S1, and CC‐S2. Gene associated with cartilage formation and development were listed on the right. B) Temporal gene expression of representative markers of CSPC, proliferation, and cartilage ECM in P0, CC‐S1, and CC‐S2. Data are means ± SEM. C) GO enrichment analysis comparison between CC‐S1 and P0. D) Analysis of Kyoto Encyclopedia of Genes and Genomes (KEGG) pathway identifying DEGs of CC‐S1 and P0. E) Heatmaps of genes related to metabolism and inflammatory response in P0 and CC‐S1. F) Schematic diagram showing representative biological events during the formation of hyperplastic macro‐cartilage.

To zoom in on the intermediate programs during the CC induction, we analyzed the transcription profiles of chondrocytes at three different stages (P0, CC‐S1, and CC‐S2) (**Figure** **5**A).

Despite the large overlap in transcripts, the volcano plots of DEGs indicated that the samples at the three‐time points had distinct phenotypes (Figure S6A–C, Supporting Information). We analyzed cartilaginous characteristics by examining associated genes. P0 demonstrated the highest level of typical CSPC markers (e.g., *NOTCH1*, *VCAM*, *CD44*), whereas CC‐S2 had the lowest level (Figure 5A,B). CC‐S1 possessed the highest expression of genes related to cell proliferation (e.g., *MKI67*, *BUB1*, *MYBL2*), but a reduced level of genes encoding cartilage functional ECM (e.g., *COL2A1*, *DCN*) when compared with P0, indicating slight dedifferentiation in CC‐S1 (Figure 5A,B). Otherwise, CC‐S2 presented the highest expression of typical cartilage ECM markers (e.g., *COL2A1*, *ACAN*, *MATN3*, *COMP*) (Figure 5A,B). These results revealed that the step‐wise CC culture achieved macro‐cartilage expansion by mainly inducing proliferation in stage 1 and chondrogenic differentiation in stage 2.

To better understand the crucial programs in CC culture, we identified GO terms in biological processes to characterize the DEGs of CC‐S1 and P0. In addition to proliferation‐related terms, we also observed that genes of the oxidation‐reduction process and electron transport chain were enhanced in CC‐S1, compared with P0 (Figure 5C). Likewise, according to the Kyoto Encyclopedia of Genes and Genomes (KEGG) pathway analysis, up‐regulated genes in CC‐S1 were related to a series of metabolic activities (e.g., carbon metabolism, biosynthesis of amino acids, glutathione metabolism, glycine, serine, and threonine metabolism, glycolysis/ gluconeogenesis) (Figure 5D,E). Consistent with our previous study, early dedifferentiated chondrocytes with enhanced metabolism still possessed the potential to be re‐differentiated,^[^ ^25^ ^]^ thus CC‐S2 acquired typical cartilage characteristics in stage 2 (Figures 3C,F and 5A,B and Figure S6D, Supporting Information). On the other hand, because dedifferentiation finely represents cell plasticity, we noted that CC‐S1 also highly expressed genes that drove early reprogramming.^[^ ^26^ ^]^ These genes included *LIN28A*, *SALL4*, and *WNT4*, which were shown to promote limb bud development and initiate regeneration (Figure S6E, Supporting Information).^[^ ^27^ ^]^ Furthermore, we detected an obvious down‐regulation of the inflammatory response‐related genes in CC‐S1 (Figure 5C). A substantial number of typical inflammation‐related genes (e.g., *IL1A*, *CCL4*, *CXCL8*, *NFKB1*) were significantly suppressed after stage 1 culture (Figure 5E). This suppression of inflammation was reported as an important trigger for the acquisition of plasticity.^[^ ^26^ ^]^ Taking these results together, CC‐S1 was considered as an intermediate plastic stage.

In conclusion, stage 1 of CC culture was characterized by enhanced proliferation and metabolism, while stage 2 was characterized by chondrogenic differentiation (Figure 5F). Thus, the entire formation process of CC macro‐cartilage displayed a proliferation‐to‐differentiation program. In addition, CC‐S1 possessed the feature of a plastic stage, implying a potential trigger of efficient proliferation in our customized system.

### 2.7 CC Macro‐Cartilage Maintains Lineage Phenotype and Promotes Cartilage Repair In Vivo

**Figure 6:**
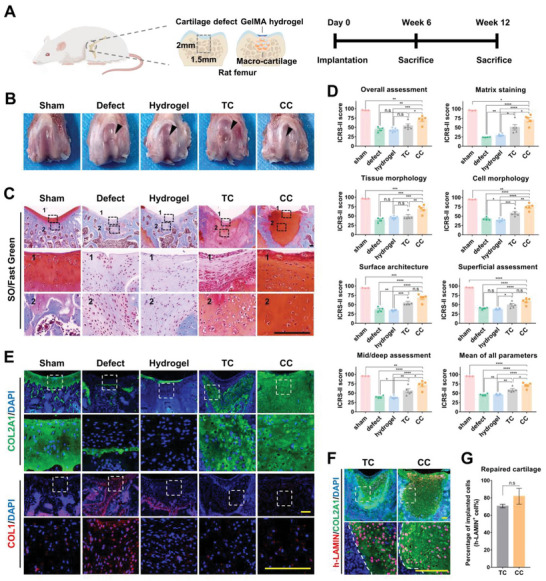
CC macro‐cartilage maintains lineage phenotype and promotes cartilage repair in vivo. A) Schematic diagram of macro‐cartilage implantation in critical‐sized cartilage defects. B) Overall repair of cartilage defects 12 weeks after implantation. C) Representative images of repaired cartilage stained by SO/fast green. Scale bar: 200 µm. D) ICRS‐II scoring for histological assessment of repaired cartilage (*n* = 3–6, one‐way ANOVA followed by Tukey's multiple comparison test). E) Immunofluorescence staining of repaired cartilage for detection of COL2A1 and COL1. Scale bar: 200 µm. F) Immunofluorescence staining of implanted chondrocyte for detection of human LAMIN in repaired cartilage. Scale bar: 200 µm. G) Quantification of chondrocytes derived from implanted macro‐cartilage in repaired cartilage (*n* = 3, unpaired two‐tailed Student's *t*‐tests). All data were mean ± SEM. n.s *p* ≥ 0.05, *p < 0.05, **p < 0.01, ***p < 0.001, ****p < 0.0001.

To further examine their in vivo function, the CC and TC tissues were implanted into a full‐thickness cartilage defect model of critical size (1.5 mm in diameter) in rats (**Figure** **6**A).^[^ ^28^ ^]^ The implants were sealed with GelMA hydrogel. Defects sealed with GelMA hydrogel only (hydrogel group) and defects without treatment (defect group) were chosen as controls.

The defect of the CC group was filled by neo‐cartilage after 6 weeks (Figure S7A,B, Supporting Information). At 12 weeks postoperatively, smooth surface integration with the host cartilage was observed (Figure 6B). SO/fast green staining further revealed that the repaired tissue of the CC group possessed a GAG‐rich ECM with lacunae of spherical cells (Figure 6C). In the other groups, the repaired regions presented an incomplete filling (Figure 6B). The repaired tissue in the TC group had less GAG content, while only fibrous tissue was observed in the defect and hydrogel group, as defects of this critical size in rats could not heal spontaneously (Figure 6C). In addition, compared with the TC group, the CC group had a relatively weaker inflammatory response (Figure S8A,B, Supporting Information). The ICRS‐II scoring system was used to quantify the outcome of joint repair.^[^ ^29^ ^]^ Notably, the CC group obtained significantly higher scores than the other groups in matrix staining, tissue morphology, overall assessment, etc., and possessed the highest score in the mean of all parameters (Figure 6D and Figures S7C and S8B, Supporting Information). By immunofluorescence staining, a high COL2 expression and low COL1 expression were detected in the repaired regions of both the CC and TC group (Figure 6E). These findings indicated that CC macro‐cartilage displayed better performance in cartilage repair.

Moreover, the COL2^+^ repaired tissues of the CC and TC groups contained (81.76 ± 9.13)% and (70.57 ± 1.95)% of human LAMIN‐positive cells, respectively (Figure 6F,G). These data demonstrated that the neo‐cartilage tissue of the CC and TC group was mainly formed by implanted human chondrocytes. It also indicated that macro‐cartilage could retain its functional phenotype in the joint defect.

Together, due to the maintenance of a GAG‐rich and homogeneous matrix in situ, CC macro‐cartilage could serve as a promising graft to promote the repair of articular cartilage in vivo.

## 3 Discussion

The transplantation of cartilage analogs provides a promising treatment for cartilage damage. Nevertheless, most strategies hardly generate sufficient off‐the‐shelf grafts with sustainable availability and controllable quality. Here, we achieved the development of hyperplastic human macromass cartilage that can proliferate and retain typical cartilage phenotype in 3D culture, using an applicable cell resource and a tailored culture condition.

The current fabrication of cartilage analogs hardly meets the demand for clinical use. Due to the contradiction between cell proliferation and phenotype maintenance, the 3D‐cultured tissue was usually presented with a limited size and even necrotic cores.^[^ ^7^, ^30^ ^]^ In this study, we yielded large‐size human polydactyly‐derived macro‐cartilage, featured with a high proliferative ability and a typical ECM‐rich phenotype. The customized macro‐cartilage originated from 2 × 10^5^ primary chondrocytes. Its diameter kept growing over time and reached ≈3.25 mm. Compared with cartilage tissues previously reported which only had a diameter of less than 1.5 mm,^[^ ^8^, ^9^ ^]^ our strategy could greatly reduce the number of grafts needed to fill a cartilage defect. For example, in the animal study, the estimated dose required for effective healing using our macro‐cartilage was only 1/4 of that using Chondrospheres.^[^ ^6^, ^31^ ^]^ Notably, unlike the hollow tissues reported in previous studies,^[^ ^8^, ^9^ ^]^ our macro‐cartilage exhibited high cellular viability in the center without a necrotic core. The structure demonstrated abundant and homogeneous cartilage ECM, supporting that CC macro‐cartilage can form superior cartilage tissue for articular regeneration. Therefore, we provide a novel strategy for clinical cartilage regeneration that can satisfy both quantity and quality demands in cartilage tissue fabrication.

In our strategy, the choice of cell resource and optimization of culture conditions were both crucial for the final output. Cellular plasticity refers to the potential of cells to change their fate or phenotype.^[^ ^32^ ^]^ It is important in cell expansion as allowing cells to re‐enter the cell cycle and undergo de‐differentiation followed by re‐differentiation.^[^ ^33^ ^]^ We hypothesized that using cells with high plasticity might meet the requirements of both quantity and quality in macro‐cartilage production. Therefore, we adopted a unique juvenile chondrocyte resource that was applicable and supposed to have a high proliferative and chondrogenic capacity.^[^ ^9^, ^11^ ^]^ As expected, polydactyly chondrocytes were indeed able to re‐express high levels of cartilage lineage markers after a 14.59‐fold expansion. The high expression of CSPC markers in our RNA‐seq data suggested that the original cells may contain chondrocyte progenitor subpopulations, which were documented to possess stem cell features but have a more specific chondrogenic commitment. This result was consistent with unpublished single‐cell RNA‐seq data of polydactyly cartilage (http://db.cngb.org/cnsa/project/CNP0001400_c26d3b0f/reviewlink/). It may explain the superior plasticity of polydactyly chondrocytes in macro‐tissue induction including both proliferative capacity and the typical chondrogenic potential. Although the specific expanded subpopulation needs to be further investigated, we still demonstrated a proof‐of‐concept study to solve the paradox in cartilage regeneration by considering the intrinsic property of chondrocyte resources.

Besides, our customized culture also played an important role in maximizing the plasticity of polydactyly chondrocytes. The tailor‐made induction strategy supplied a suitable microenvironment and contributed to a better performance in macro‐cartilage fabrication. In comparison to typical culture, CC conducted an enhanced expansion (2.57 times TC) and produced a preferable tissue structure (large and intact). Interestingly, our data showed that CC induced a step‐wise procedure through an intermediate stage shown with a higher expression of proliferative markers and metabolic genes. In other reports, a high metabolic level is one of the features of the cells with latent regenerative potential or expansion capacity.^[^ ^24^, ^34^ ^]^ Our previous study also indicated that metabolic activation benefited cell adaption during in vitro growth, supporting that CC‐cells survived in the hypoxic central of macro‐tissue.^[^ ^25a^ ^]^ Additionally, early‐stage cells in our procedure displayed a notable enrichment of genes that were documented to be expressed at early reprogramming to pluripotency (and this stage was termed as the intermediate plastic state).^[^ ^26^ ^]^ Similarly, CC macro‐tissue exhibited a relatively low level of inflammatory markers. It was previously recorded that the induction of this plastic state with suppressed inflammation was the key to inducing dedifferentiation and triggering regeneration‐like events.^[^ ^26^ ^]^ Taken together, these findings suggested that the conditions of CC culture induced an intermediate stage when the plasticity of polydactyly chondrocytes could be activated. Hence, the polydactyly chondrocytes were able to undergo a dedifferentiation‐to‐redifferentiation process to grow into intact ECM‐rich tissue. Although the critical components and molecular mechanisms need a deeper investigation, this study still highlighted that an optimized culture could be used to manipulate cell plasticity and create new cell states. It provides a promising platform to generate functional tissue grafts in regenerative medicine.

Allotransplantation using natural cartilage tissue and chondrocytes has been applied for decades, and postoperative immunosuppressive therapy is not required in previous cases.^[^ ^3^, ^35^ ^]^ However, there are still some safety concerns that need to be addressed. Although evidences showed that polydactyly chondrocytes lacked the expression of MHC‐II, the immunogenicity of macro‐cartilage may change during the in vitro culture procedure and needs to be evaluated before transplantation.^[^ ^11^ ^]^ Additionally, it is still unknown if macro‐cartilage can stimulate the immune responses of the hosts, as immunosuppressants were administered in this study. It will be necessary to assess whether CC macro‐cartilage has a better repair outcome than TC cartilage in the absence of immunosuppressants in future preclinical studies.

In conclusion, we achieved a 3D customized expansion of hyperplastic human macro‐cartilage by regulating cellular plasticity. This study provides a new therapeutic strategy to promote cartilage regeneration and insights for understanding and regulating cell intrinsic regenerative potential.

## 4 Experimental Section

### Chondrocytes Isolation

Human chondrocytes were isolated from finger or toe cartilage after polydactyly resection surgery. This experiment had been approved by the ethics committee of the Children's Hospital of Zhejiang University School of Medicine (2020‐IRB‐007), and all individuals provided full written informed consent before the operative procedure. Briefly, fresh cartilage tissue was washed twice with PBS, cut into slices, and digested with 0.2% collagenase type II (Gibco) in DMEM/F12 (Gibco) at 37 °C overnight. The digestion solution was filtered using a 40 µm filter to remove the minced tissue, and centrifuged at 1500 rpm for 5 min. After the supernatant was aspirated, the primary chondrocytes were collected for subsequent experiments.

### Screen for Serum‐Free Culture Condition

Primary chondrocytes were seeded in a 96‐well plate and cultured in different media formed by DMFM/F12 supplemented with different combinations of candidate components (Table S2 and Figure S2A, Supporting Information). The medium was refreshed on day 3. The effect was assessed by the area of cell clusters observed under the microscope (Olympus) on day 6. After determining the optimal combination, eight additives were added separately to culture primary chondrocytes for further screening and optimization (Table S3 and Figure S2B, Supporting Information). The medium was refreshed on day 3, and then the effects were also assessed on day 6.

### CC Culture

Primary chondrocytes were cultured in the customized SFM (CM) and maintained for 6 days. The CM was composed of DMEM/F12 (Gibco), 2% B27 (Gibco), 30 ng mL^−1^ bFGF (PeproTech), and 10 ng mL^−1^ TGF*β*3 (PeproTech). The medium was refreshed every 3 days during stage 1. After 6 days, the medium was changed to a chondrogenic differentiation medium for another 21 days. The medium was composed of H‐DMEM (Gibco), 10 ng mL^−1^ TGF*β*3 (PeproTech), 10^−7^ m dexamethasone (Sigma), 1% ITS (Gibco), 50 mg mL^−1^ ascorbic acid (Sigma), and 1 mm sodium pyruvate (Gibco). This medium was also changed every 3 days during stage 2. As a control, chondrocytes were cultured in a typical serum‐containing medium (TM) which was composed of DMEM/F12 (Gibco) and 10% FBS (Gibco) for 6 days. Then, the medium was changed to a chondrogenic differentiation medium for another 21 days. The frequency of medium refresh was synchronized with the CC group.

### Viability Assay

Cell viability was assessed qualitatively with cell counting Kit‐8 (CCK‐8, Dojindo) on days 1, 3, 6, 9, 13, 20, and 27. In brief, the medium was changed to DMEM/12 supplemented with 10% CCK‐8 reagent and incubated at 37 °C for 2 h in the dark. The optical density at 450 nm (OD 450 nm) was measured using a multiwell plate reader (Bio‐Rad).

### Proliferating Cell Visualization and Assay

The proliferating chondrocytes were labeled with EdU Cell Proliferation Kit (Cellorlab) on days 1, 3, 6, 9, 13, 20, and 27. Briefly, 10 µm EdU was added to chondrocytes for 4 h. Cells were fixed in 4% paraformaldehyde (PFA), EdU was detected with a Click reaction mixture containing Azide, and cell nucleus were stained with Hoechst 33 342 (5 µg mL^−1^). The results were visualized with confocal microscopy (Nikon) and quantified by the percentage of EdU/Hoechst‐positive cells.

### Cytoskeleton and Nuclei Visualization

After fixed in 4% PFA, cells were treated with 5 µg mL^−1^ 4′,6‐diamidino‐2‐phenylindole (DAPI) (Beyotime) and 100 nm Acti‐Stain 488 Phalloidin (Cytoskeleton) in the dark for 30 min. The cell nucleus was labeled by DAPI, and the cytoskeleton (F‐actin) was stained by Phalloidin. The staining was visualized with confocal microscopy (Olympus).

### Construct Microtissues in a High Through‐Put Platform

Agarose microwell inserts were prepared as previously described.^[^ ^20b^ ^]^ Briefly, 3% (w/v) Agarose (Sigma) was poured onto a polydimethylsiloxane (PDMS) customized mold (RDMICRO) containing pillars with a diameter of 200 µm. After solidified, the agarose microwell was separated, punched out with an area of 1.8 cm^2^, and placed in 24 well plates. Each insert contained about 1000 microwells and they were immersed in PBS and sterilized with UV light for 30 min. Primary chondrocytes were seeded with a concentration of 2 × 10^4^ cells per microwell to obtain about 20 cells per microwell. The chondrocytes were cultured in CM/TM and the medium was changed every 3 days. On day 6, the medium was switched to a chondrogenic differentiation medium for 21 days and refreshed every 3 days during this period.

### Macro‐Tissues Construction

To construct large‐size tissues, 2 × 10^5^ primary chondrocytes were seeded into a 15 mL conical tube containing CM/TM and then centrifuged for 5 min at 1200 rpm to form a high‐cell density pellet. The medium was changed every 2 days. On day 6, the medium was changed to chondrogenic differentiation medium for 21 days and refreshed every 3 days during this period.

### Bulk RNA Sequencing

Total RNA was extracted from samples using Trizol (Takara) according to manual instructions. Library construction and RNA‐seq were performed by the BGI‐China using the BGISEQ‐500 platform. The sequencing raw data were filtered with SOAPnuke (v1.5.2), and the clean reads were mapped to a reference genome using HISAT2 (v 2.0.4). After alignment using Bowtie (v 2.2.5), the expression level of each gene was calculated by RSEM (v 1.2.8), and differential expression analysis was performed using DESeq2 (v 1.4.5) with the parameters fold change ≥2 and adjusted *p*‐value ≤0.05. The sequencing data analysis, including heatmap clustering, principal component analysis (PCA), sample correlation, Venn diagram creation, GO analysis, GSEA analysis, and KEGG pathway analysis, were performed using BGI Dr. Tom 2.0.

### Cell Immunofluorescence Staining

For the cell immunofluorescence assay, cells were fixed in 4% PFA, permeabilized with 0.3% Triton X‐100 for 15 min, and blocked with 1% bovine serum albumiin (BSA) for 30 min. After that, cells were incubated with primary antibodies against COL2A1 (1:100, Santa Cruz, sc‐52658), SOX9 (1:100, Abcam, ab76997), and COL1 (1:200, Affinity, AF7001) overnight at 4 °C. Then, cells were washed and incubated with secondary antibodies Alexa Fluor 488 (1:1000, Invirogen, A21202) or Alexa Fluor 546 (1:500, Invirogen, A11035) for 2 h in the dark. After counterstaining with DAPI, images were taken by confocal microscopy (Olympus) and fluorescence was quantified by ImageJ software.

### In Vivo Implantation

10‐week‐old male Sprague–Dawley rats were used for a model of full‐thickness cartilage defect. A critical‐sized cylindrical defect (1.5 mm in diameter and 2 mm in depth) was created on the trochlear groove of the distal femurs of these rats with a drill bit (1.5 mm in diameter).^[^ ^28^ ^]^ The defects were randomly managed with one of the following methods: no treatment (defect group); filling with GelMA hydrogel (hydrogel group); filling with CC macro‐cartilage and GelMA hydrogel (CC group); filling with TC cartilage and GelMA hydrogel (TC group). The sham‐operated group was used as a positive control. To avoid immune rejection, rats were given daily administration of cyclophosphamide (4 mg kg^−1^ body mass, ENDOXAN) from 24 h prior to grafting until the end of the experiment.^[^ ^36^ ^]^ The rats were sacrificed at weeks 6 (sham, *n* = 3; defect, *n* = 6; hydrogel, *n* = 5; TC, *n* = 6; CC, *n* = 5) and 12 (sham, *n* = 3; defect, *n* = 4; hydrogel, *n* = 6; TC, *n* = 5; CC, *n* = 5) postoperatively for subsequent histological analysis. The animal experiment was approved by Zhejiang University Ethics Committee (ZJU20210284).

### Histological Staining and Analysis

The cultured tissues were fixed in 4% PFA overnight at room temperature (RT), dehydrated through an alcohol gradient, and embedded in paraffin blocks. Rat joint samples were harvested and fixed in 4% PFA for over 48 h at RT. Then, the joint samples were decalcified in EDTA/PBS (pH 7.5) for 8 weeks, dehydrated, and embedded in paraffin. The cultured tissues and joint samples were sectioned at 6 and 7 µm, respectively, using a microtome (Leica).

For Safranin‐O/fast green staining, after deparaffinized and hydrated, paraffin sections were stained with fast‐green (Sigma) for 8 min and Safranin‐O (Sigma) for 8 min at RT. Micrographs of reconstructed cartilage were blindly scored by five independent reviewers following the ICRS‐II scoring system.^[^ ^29^ ^]^

For immunofluorescence staining, paraffin sections were deparaffinized, hydrated, and then unmasked with 0.05% (w/v) trypsin/EDTA for 30 min at 37 °C. Sections were permeabilized with 0.3% Triton X‐100 for 15 min and blocked with 1% BSA for 30 min. After that, sections were incubated with primary antibodies against COL2A1 (1:100, Santa Cruz, sc‐52658), ACAN (1:200, Abcam, ab36861), COL6 (1:250, Abcam, ab6588), PRG4 (1:100, Abcam, ab28484), and COL1 (1:200, Affinity, AF7001) overnight at 4 °C. Then, sections were washed and incubated with secondary antibodies Alexa Fluor 488 (1:1000, Invirogen, A21202) or Alexa Fluor 546 (1:500, Invirogen, A11035) for 2 h in the dark. After counterstaining with DAPI, images were taken by confocal microscopy (Olympus) and fluorescence was quantified by ImageJ software.

### Statistical Analysis

All experiments were independently performed at least three times. The results are expressed as means ± SEM. For comparisons of two groups, the data were analyzed by unpaired two‐tailed Student's *t*‐tests. For comparisons of multiple groups, the data were analyzed by one‐way analysis of variance (ANOVA) with Tukey's test or Dunnett's multiple comparisons test. Results were considered statistically different for *p*‐values lower than 0.05 (n.s *p* ≥ 0.05, *p < 0.05, **p < 0.01, ***p < 0.001, ****p < 0.0001). All analyses were performed using GraphPad Prism 7 software.

## Conflict of Interest

The authors declare no conflict of interest.

## Author Contributions

Y.W., Y.C., and W.W. contributed equally to this work. Y.W., Y.C., and H.O. conceived and designed the study. Y.W. performed the experiments and sequencing data analysis. W.W., H.Z., H.S., H.L., and X.C. helped with the establishment of a culture system. Y.C., Z.P., W.J., X.S., and J.H. helped with RNA‐seq data analysis. X.Z. and Y.L. helped with the animal experiments. Y.X. helped with the histological analysis. J.C. and W.W. helped with the collection of human samples. Y.W., Y.C., and H.O. wrote the manuscript. H.O., Y.C., and X.Y. revised and corrected the manuscript. H.O. supervised the study. All authors edited and approved the final paper.

## Supporting information

Supporting Information

## Data Availability

The data that support the findings of this study are available in the supplementary material of this article.
