# Supplementary material for: Hyperplastic Human Macromass Cartilage for Joint Regeneration
Source: Adv Sci (Weinh). 2023 Jul 3;10(26):2301833. doi: 10.1002/advs.202301833 (PMC10502860; doi:10.1002/advs.202301833)
Supplement: Supplementary file 1 — Supporting Information [file ADVS-10-2301833-s001.pdf]

ADVANCED SCIENCE
Open Access

Supporting Information

for *Adv. Sci.*, DOI 10.1002/advs.202301833

Hyperplastic Human Macromass Cartilage for Joint Regeneration

Ya Wen, Yishan Chen, Weiliang Wu, Hong Zhang, Zhi Peng, Xudong Yao, Xianzhu Zhang, Wei Jiang, Youguo Liao, Yuan Xie, Xilin Shen, Heng Sun, Jiajie Hu, Hua Liu, Xiao Chen, Jiansong Chen and Hongwei Ouyang**

Supporting Information

Supplementary Experimental Procedures

CM ingredients verification. All ingredients were added to DMEM/F12 as positive control. By removing one ingredient and keeping others constant, corresponding impacts on cell proliferation were identified (Table S4, Figure S2C, D). Primary chondrocytes were seeded in 96-well plate and cultured in candidate medium. The medium was refreshed at day 3. The effect was assessed by the area of cell clusters observed under the microscope (Olympus) at day 6.

RNA extraction, reverse transcription, and quantitative real-time PCR. Total RNA was isolated from samples using Trizol (Takara) and transcribed into cDNA using a ReverTra Ace® qPCR RT Master Mix (TOYOBO) according to the protocols. Quantitative real-time PCR was performed on the LightCycler®480 PCR System (Roche) using TB Green® Premix Ex Taq™ (Takara). The relative expression levels of each gene were analyzed using the $2^{-\triangle\triangle Ct}$ method. The forward and reverse primers for each gene are shown in Table S5.

Scanning electron microscopy. According to previous reports, paraffin sections were deparaffinized and hydrated [1]. After sputter coated with gold particles, the samples were imaged with a scanning electron microscopy (SEM, Hitachi SU8010).

Cell viability assessment of macro-cartilage. Cell viability in macro-cartilage was assessed by Live/Dead staining and trypan blue staining.

For Live/Dead staining, macro-cartilage was incubated in 20 μᴍ Calcein AM (Dojindo) and 20 μᴍ Cellstain- PI (Dojindo) at 37°C for 30 min. After rinsed with

PBS, the stained macro-cartilage was visualized with a confocal microscope (Olympus, FV1000).

For trypan blue staining, macro-cartilage was digested into single cells and mixed with 0.4% trypan blue. The stained cells were quantified within 3 min through automated cell counter (Countstar).

Supplementary Figures

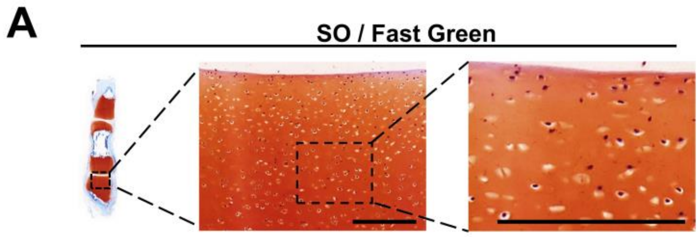

Figure S1. The polydactyly cartilage exhibits a hyaline cartilage-like phenotype.

(**A**) Representative images of polydactyly digital stained by Safranin- O (SO)/fast green. Scale bar: 200 μm.

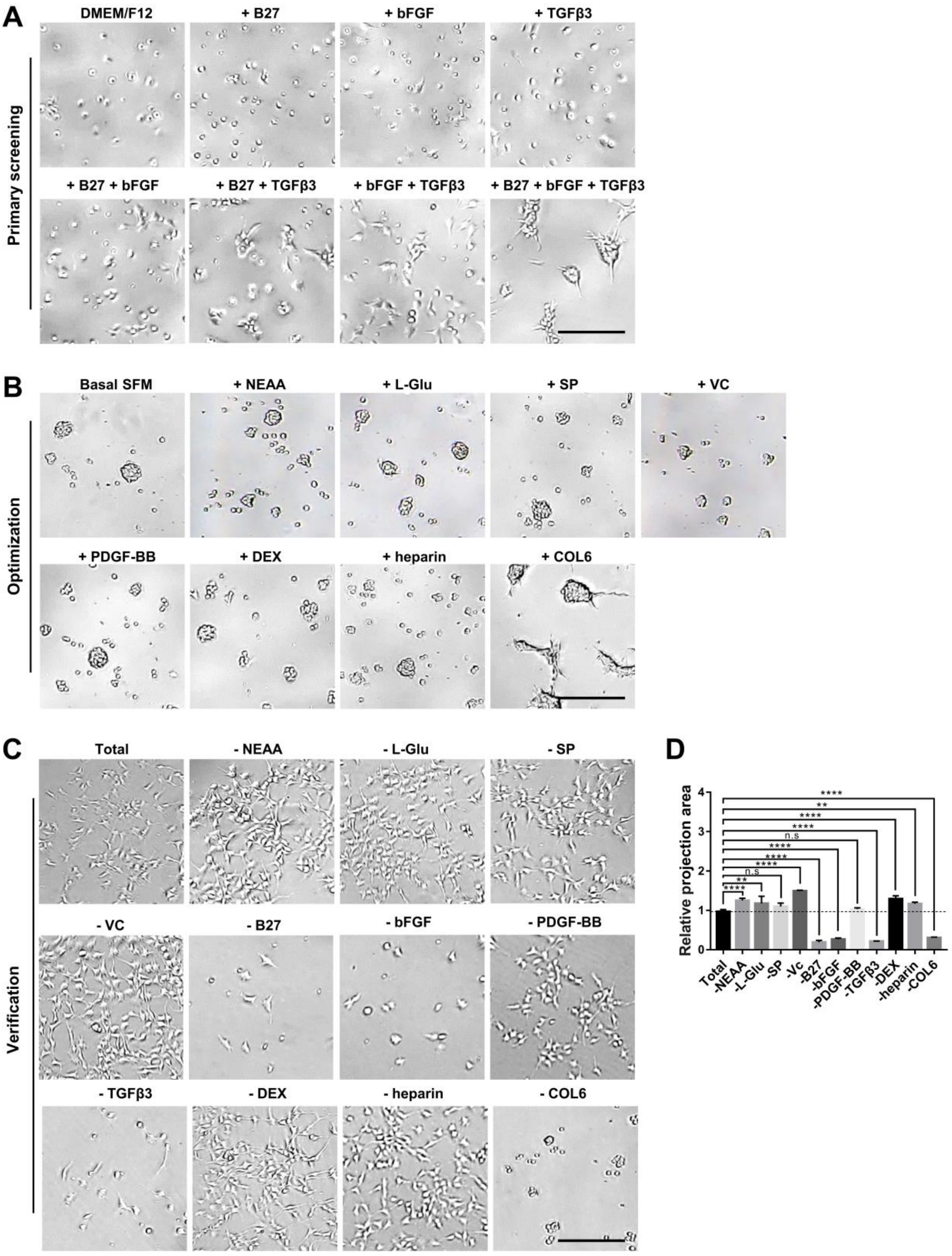

Figure S2. A screen-defined serum-free medium efficiently expands polydactyly chondrocyte *in vitro*. Related to Figure 1.

(**A-C**) Representative morphologies of primary polydactyly chondrocytes

cultured in different culture conditions. Scale bar: 200 μm. (**D**) Effect quantification of different additives (*n*=3, one-way ANOVA followed by Dunneett's multiple comparison test). All data were mean ± SEM. n.s $p \geq 0.05$, $^{**}p < 0.01$, $^{****}p < 0.0001$.

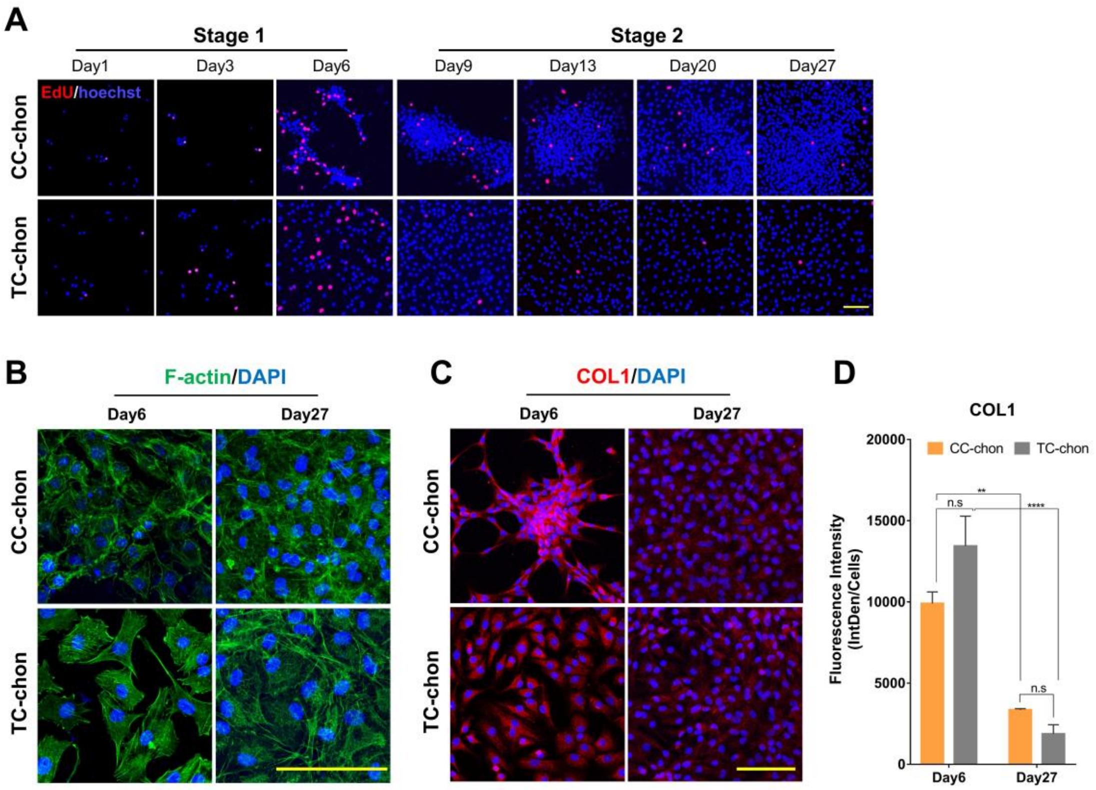

Figure S3. Characterization of CC-chons. Related to Fig1.

(**A**) Representative fluorescent images of proliferating cells (EdU) in CC and TC over time, DAPI labeled the nucleus. Scale bars: 100 μm. (**B**) Representative images of CC-chons and TC-chons stained with DAPI (nucleus) and Phalloidin (F-actin). Scale bars: 100 μm. (**C**) Representative images of immunofluorescent staining of COL1 in CC-chons and TC-chons. Scale bars: 100 μm. (**D**) Efficiency quantification of COL1 staining (n = 6, one-way ANOVA followed by Tukey's multiple comparison test). All data were mean ± SEM. n.s $p \geq 0.05$, **$p < 0.01$, ****$p < 0.0001$.

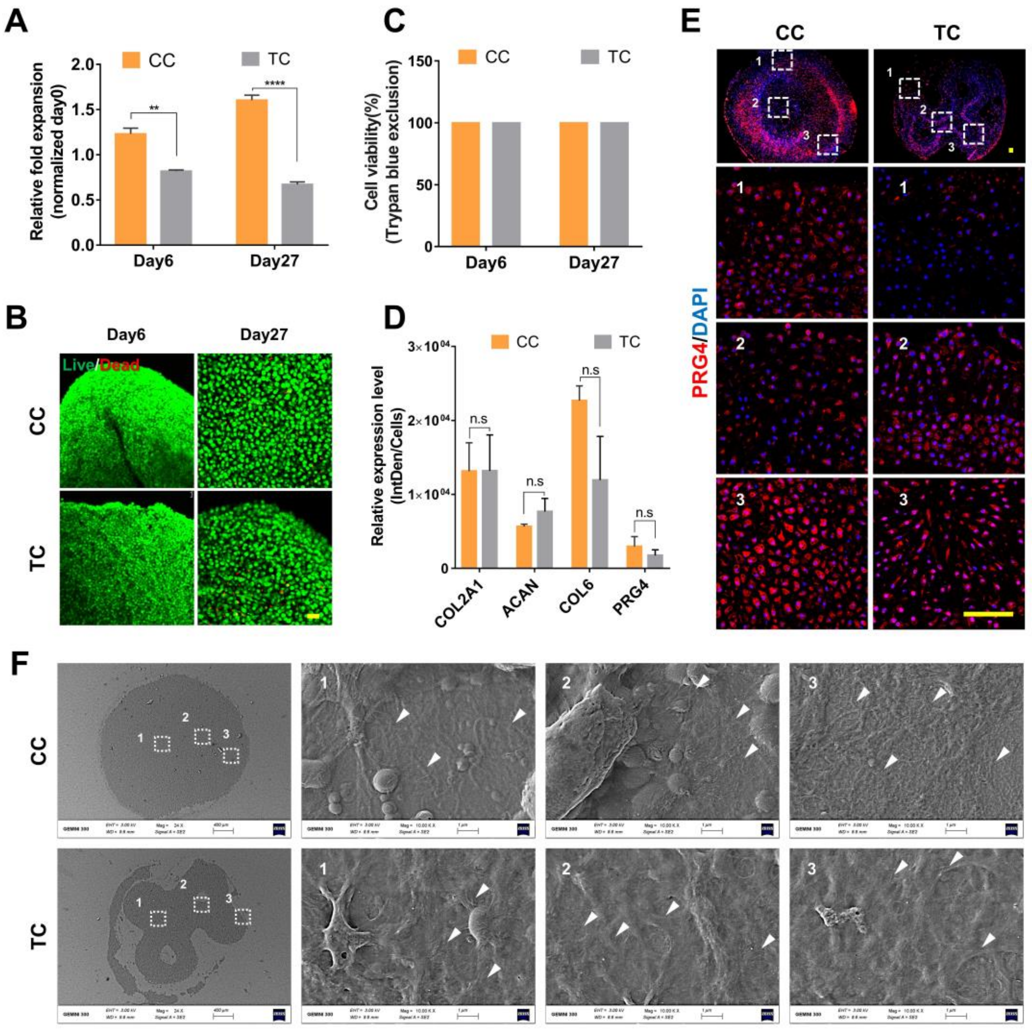

Figure S4. Characterization of macro-cartilage. Related to Fig3.

(**A**) Cumulative fold expansion in CC and TC culture at day 6 and day 27 (*n*=3, unpaired two-tailed Student's t tests). (**B**) Representative images of Live (green)/Dead (red) staining in CC and TC tissues at day 6 and day 27. (**C**) The percentage of viable cells in CC and TC tissues at day 6 and day 27, characterized by trypan blue staining (*n*=3). (**D**) Representative images of immunofluorescent staining of PRG4 in CC and TC tissues (*n*=3, unpaired two-tailed Student's t tests). (**E**) Efficiency quantification of COL2A1, ACAN, PRG4 staining in CC and TC tissues. Scale bars: 100 μm. (**F**) Representative SEM images of CC and TC tissues. All data were mean ± SEM. n.s $p \geq 0.05$, **$p < 0.01$, ****$p < 0.0001$.

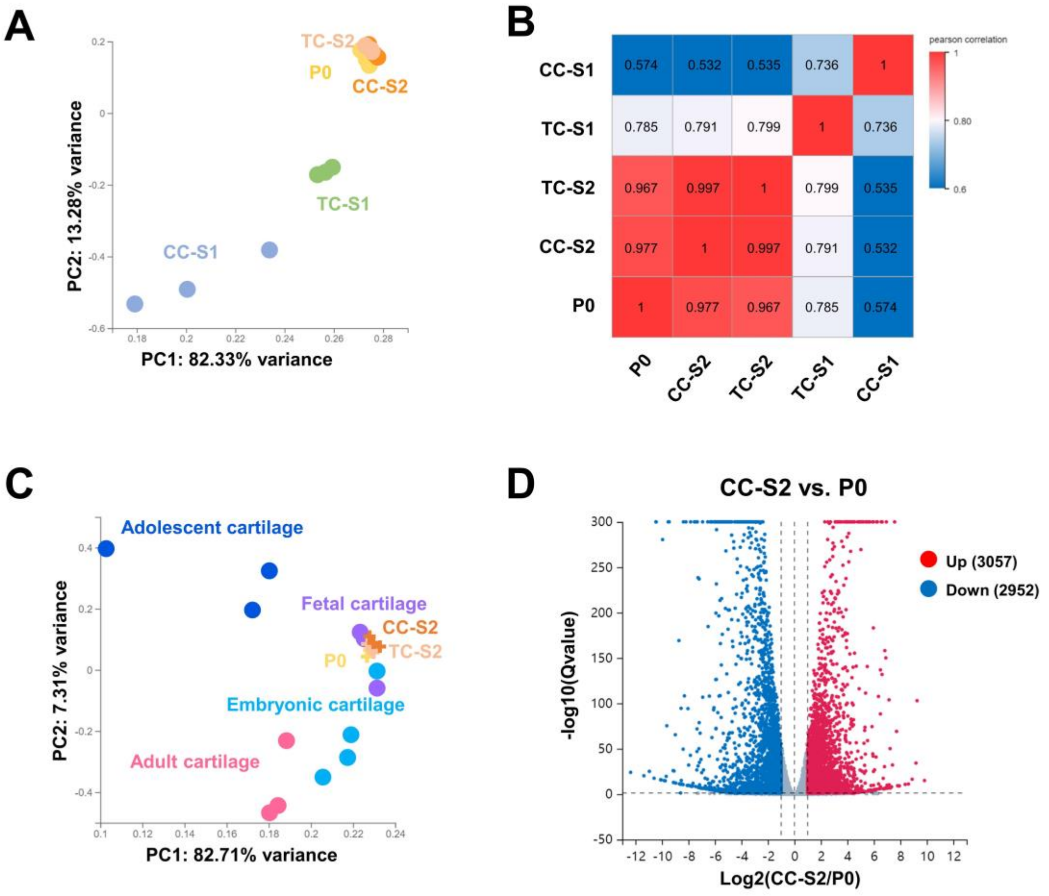

Figure S5. Overall transcriptome analysis of macro-cartilage. Related to Fig 4.

(**A**) Principal-component analysis (PCA) plot of all samples. (**B**) Correlation coefficient matrix of all samples. (**C**) Principal-component analysis (PCA) plot of P0, CC-S2, TC-S2 and human articular cartilage at different developmental stage. (**D**) Volcano plot identifying genes that are differentially expressed between CC-S2 and P0.

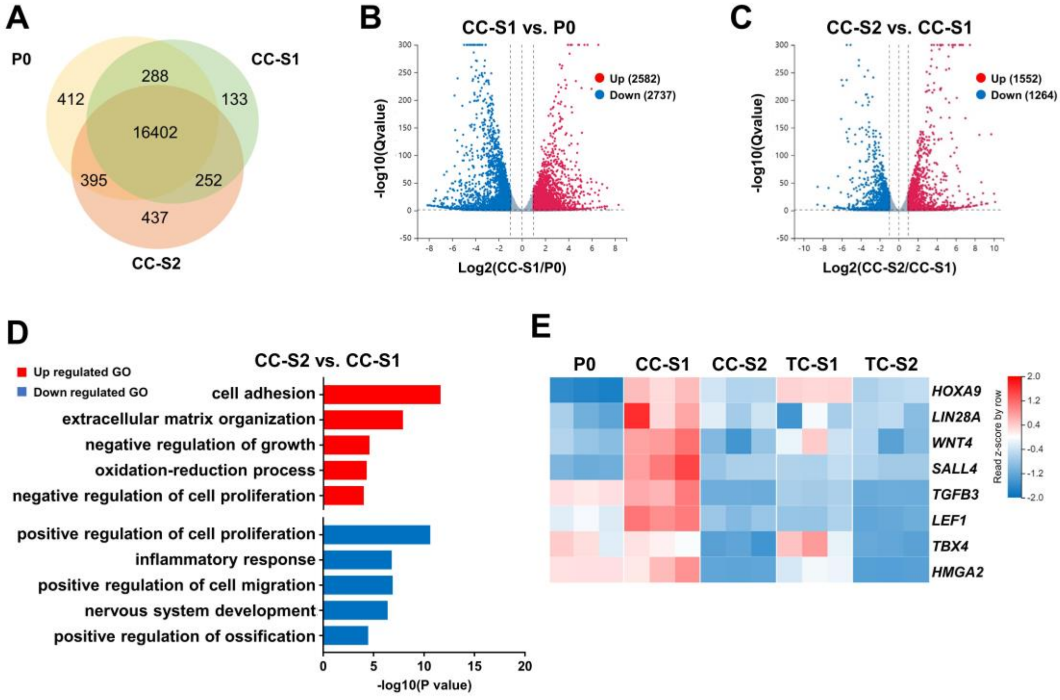

Figure S6. CC-chondrocytes acquire an intermediate plastic phenotype in Stage 1. Related to Fig 5.

(**A**) Venn diagram of the number of genes expressed in different stage of CC macro-cartilage (P0, CC-S1, CC-S2). (**B**) Volcano plots of differentially expressed genes between CC-S1 and P0. (**C**) Volcano plots of differentially expressed genes between CC-S2 and CC-S1. (**D**) GO enrichment analysis comparison between CC-S2 and CC-S1. (**E**) Heatmaps of genes drove early reprogramming.

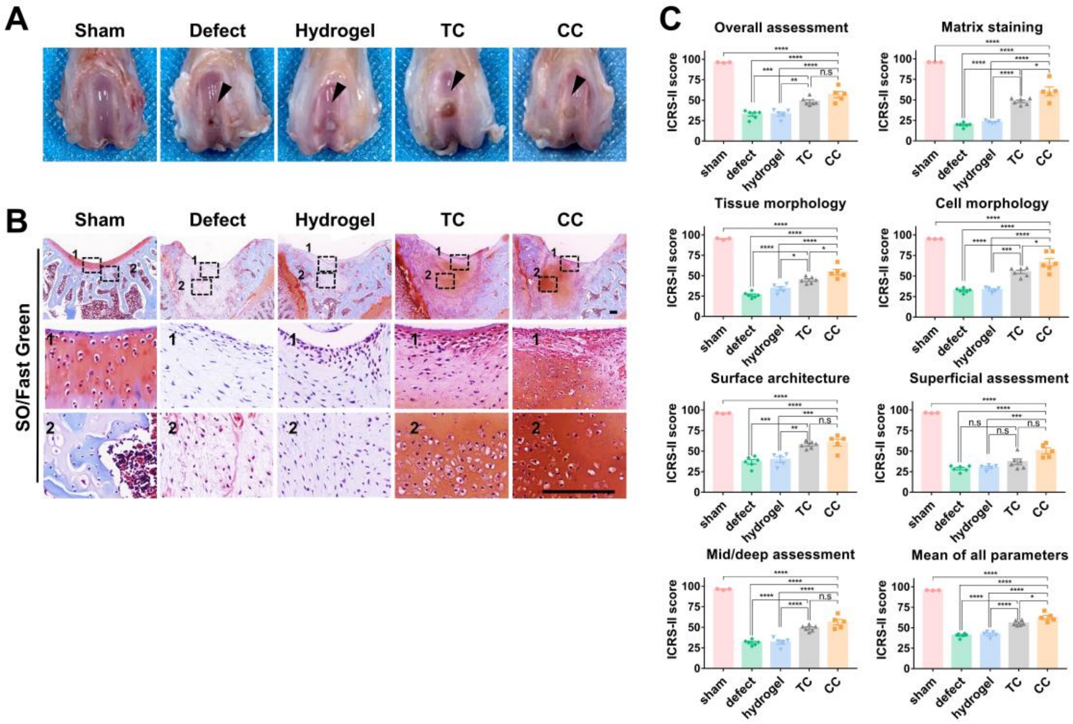

Figure S7. Evaluation of articular cartilage repair at 6 weeks post-operatively. Related to Fig6.

(**A**) Overall repair of cartilage defects 6 weeks after implantation. (**B**) Representative images of repaired cartilage stained by SO/fast green. Scale bar: 200 μm. (**C**) ICRS-II scoring for histological assessment of regenerated cartilage (*n* = 3-6, one-way ANOVA followed by Tukey's multiple comparison test). All data were mean ± SEM. n.s $p \geq 0.05$, *$p < 0.05$, **$p < 0.01$, ***$p < 0.001$, ****$p < 0.0001$.

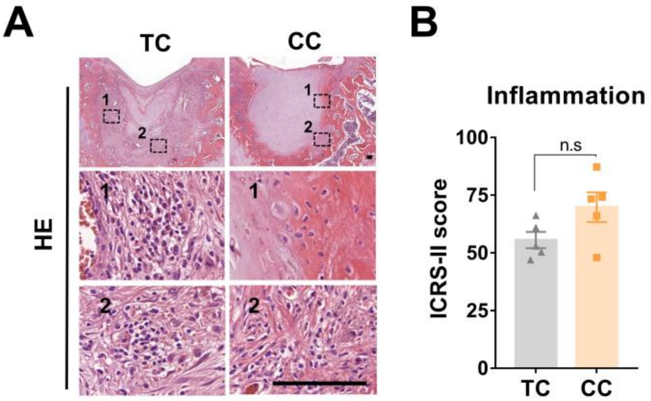

Figure S8. CC macro-cartilage elicits a relative weaker inflammatory response. Related to Fig6.

(**A**) Representative images of repaired cartilage stained by hematoxylin-eosin (HE). Scale bar: 100 μm. (**B**) ICRS-II scoring for histological assessment of regenerated cartilage (*n*=3, unpaired two-tailed Student's t tests). All data were mean ± SEM. n.s $p \geq 0.05$.

Supplementary Tables

Table S1. Donor information of human chondrocytes.

Number	Age	Sex	Experiment
1	11M26D	Female	Establishment of culture conditions
2	9M24D	Female	
3	1Y3M	Male	
4	1Y	Male	
5	1Y	Female	
6	2Y	Female	
7	1Y7M	Female	
8	1Y1M	Male	
9	5M3D	Male	
10	6M24D	Male	Characterization of CC-chons in 2D culture
11	11M	Male	
12	7M	Female	
13	1Y3M	Male	
14	4M10D	Male	*In vitro* characterization of macro-cartilage
15	1Y11M	Male	
16	7M22D	Male	
17	3M10D	Female	
18	5M12D	Female	
19	7M26D	Male	
20	13M	Female	Bulk RNA sequencing of of macro-cartilage
21	11M20D	Female	
22	4M8D	Male	
23	11M	Female	
24	6M17D	Female	
25	1Y1M	Female	

26	7M8D	Female	
27	1M9D	Male	
28	3M5D	Female	Implantation of macro-cartilage in rat joints
29	3M1D	Female	
30	6M20D	Male	
31	2M	Female	
32	5M21D	Male	
33	7M7D	Female	
34	2Y10D	Female	
35	1Y	Female	
36	1Y	Female	

Table S2. Candidate supplement in combinatory screening.

Name	Description	Source	Catalog Number	Concentration
B27	B-27™ Supplement (50×), serum free	Gibco	17504044	1×
bFGF	Fibroblast Growth Factor-basic	PeproTech	100-18B	30 ng·mL^{-1}
TGFβ3	Transforming Growth Factor-β3	PeproTech	100-36E	10 ng·mL^{-1}

Table S3. Candidate additives in optimization screening.

Name	Description	Source	Catalog Number	Concentration
NEAA	MEM Non-Essential Amino Acids Solution	Gibco	11140050	1 ×

	(100×)			
L-Glu	L-Glutamine (200mм)	Gibco	25030081	2 mм
SP	Sodium Pyruvate (100mм)	Gibco	11360070	1 mм
Vc	Ascorbic Acid	Sigma	A92902	50 μg·mL^{-1}
PDGF-BB	Platelet-Derived Growth Factor-BB	PeproTech	11-14B	20 ng·mL^{-1}
DEX	dexamethasone	Sigma	D4902	10^{-8} м
Heparin	Heparin sodium	Selleck	S1346	2 μg·mL^{-1}
COL6	Collagen 6	Abcam	ab7538	2.5 μg·mL^{-1}

Table S4. Candidate components in verification experiment.

**Name**	**Description**	**Source**	**Catalog Number**	**Concentration**
B27	B-27™ Supplement (50×), serum free	Gibco	17504044	1×
bFGF	Fibroblast Growth Factor-basic	PeproTech	100-18B	30 ng·mL^{-1}
TGFβ3	Transforming Growth Factor-β3	PeproTech	100-36E	10 ng·mL^{-1}
NEAA	MEM Non-Essential Amino Acids Solution (100×)	Gibco	11140050	1×
L-Glu	L-Glutamine (200mм)	Gibco	25030081	2 mм
SP	Sodium Pyruvate (100mм)	Gibco	11360070	1 mм

Vc	Ascorbic Acid	Sigma	A92902	50 μg·mL^{-1}
PDGF-BB	Platelet-Derived Growth Factor-BB	PeproTech	11-14B	20 ng·mL^{-1}
DEX	dexamethasone	Sigma	D4902	10^{-8} м
Heparin	Heparin sodium	Selleck	S1346	2 μg·mL^{-1}
COL6	Collagen 6	Abcam	ab7538	2.5 μg·mL^{-1}

Table S5. Primer sequences for real-time quantitative PCR analysis.

**Gene**	**Forward (5'-3')**	**Reverse (5'-3')**
COL2A1	GTCTGTGACACTGGGACTGT	TCTCCGAAGGGGATCTCAGG
ACAN	CTGCAGACCAGGAGGTATGTGA	GTTGGGGCGCCAGTTCTCAAAT
SOX9	GGCGGAGGAAGTCGGTGAAGAA	GCTCATGCCGGAGGAGGAGTGT
FGF2	AGAAGAGCGACCCTCACATCA	CGGTTAGCACACACTCCTTTG
MMP13	ATGCAGTCTTTCTTCGGCTTAG	ATGCCATCGTGAAGTCTGGT
COL1A1	CGATGGATTCCAGTTCGAGTAT	CATCGACAGTGACGCTGTAGG
ADAMTS5	ATCACCCAATGCCAAGG	AGCAGAGTAGGAGACAAC
MATN3	TCTCCCGGATAATCGACACTC	CAAGGGTGTGATTCGACCCA
GAPDH	TGACGCTGGGGCTGGCATTG	GGCTGGTGGTCCAGGGGTCT

Reference

[1] G. N. Hall, W. L. Tam, K. S. Andrikopoulos, L. Casas-Fraile, G. A. Voyiatzis, L. Geris, F. P. Luyten and I. Papantoniou, *Biomaterials* **2021**, *273*, 120820.
